# Supplementary material for: Incidence and risk factors of COVID-19 associated pneumothorax
Source: PLoS One. 2022 Aug 5;17(8):e0271964. doi: 10.1371/journal.pone.0271964 (PMC9355189; doi:10.1371/journal.pone.0271964)
Supplement: S1 File — List of inclusion and exclusion criteria for tocilizumab therapy in our institution. (DOCX) [file pone.0271964.s001.docx]

**Our institution guidance for Tocilizumab therapy:**

# **Inclusion criteria** are all the following: 1) Laboratory confirmed SARS-CoV-2. 2) ≤3 days from hospitalization due to SARS-CoV-2. 3) Received ≥24 hours of steroid therapy for SARS-CoV-2 and continues to worsen in respiratory status. 4) ≤24 hours from initiating respiratory (HFNC >30L/min and FiO2 >0.4, NIV or MV) or cardiovascular organ support (any vasopressor or inotrope). 5) Absence of other primary causes of respiratory desaturation 6) CRP ≥75 mg/L (must be resulted within 24 hours prior to tocilizumab use and after ≥ 24 hours of steroids. # **Exclusion criteria** are any of the following: 1) Patient on standard nasal cannula oxygen, nonrebreather, and/or HFNC ≤30 L/min. 2) Patients with recent use of other immunomodulating biologics. 3) ALT or AST >105 x ULN. 4) High risk for GI perforation 5) Uncontrolled, serious non-COVID-19 infection. 6) ANC <1000 cells/mL. 7) Platelets <50,000 cells/mL. 8) Patient on hospice/comfort care. 9) Patient unlikely to survive more than 24 hours from screening or have coded during admission prior to tocilizumab.

**Our Institution guidance for anticoagulation therapy**

ALL COVID 19 (+)/PUI patients without contraindications will be started on the new Enoxaparin prophylaxis protocol:

•Enoxaparin 0.5 mg/kg/dose subcutaneously q 12 hrs (maximum dose 80 mg q 12 hrs) for GFR > 30 ml/min/1.73 m2, and same dose on q 24 h interval if GFR < 30 ml/min/1.73 m2

•High risk patients (D-Dimer > 3 mg/L) will receive full anticoagulation:

•Enoxaparin 1 mg/kg/dose subcutaneously q 12 hrs (maximum dose 150 mg q 12 hrs) for GFR > 30 ml/min/1.73 m2, and same dose on q 24 h interval if GFR < 30 ml/min/1.73 m2
